# Randomised trial of a decision aid and its timing for women being tested for a BRCA1/2 mutation

**DOI:** 10.1038/sj.bjc.6601525

**Published:** 2004-01-20

**Authors:** M S van Roosmalen, P F M Stalmeier, L C G Verhoef, J E H M Hoekstra-Weebers, J C Oosterwijk, N Hoogerbrugge, U Moog, W A J van Daal

**Affiliations:** 1 Joint Center for Radiation Oncology Arnhem-Nijmegen (RADIAN), University Medical Center Nijmegen, Nijmegen, The Netherlands; 2 Medical Technology Assessment, University Medical Center Nijmegen, Nijmegen, The Netherlands; 3 Department of Medical Psychology, University Hospital Groningen, Groningen, The Netherlands; 4 Department of Medical Genetics, University Hospital Groningen, Groningen, The Netherlands; 5 Department of Human Genetics and Hereditary Cancer Clinic, University Medical Center Nijmegen, Nijmegen, The Netherlands; 6 Department of Clinical Genetics, University Hospital Maastricht, Maastricht, The Netherlands

**Keywords:** decision aid, randomised trial, BRCA1, BRCA2

## Abstract

The aim of the study was to evaluate the impact of a decision aid (DA) and its timing in women being tested for a BRCA1/2 mutation. Women with and without a previous history of cancer were included after blood sampling for genetic testing. The DA consisted of a brochure and video providing information on screening and prophylactic surgery. To evaluate the impact of the DA, women were randomised to the DA group (*n*=184), receiving the DA 2 weeks after blood sampling, or to the control group (*n*=184). To evaluate the impact of timing, mutation carriers who had received the DA *before* the test result (*n*=47) were compared to mutation carriers who received the DA *after* the test result (*n*=42). Data were collected on well-being, treatment choice, decision and information related outcomes. The impact of the DA was measured 4 weeks after blood sampling. The impact of timing was measured 2 weeks after a positive test result. The DA had no impact on well-being. Regarding decision related outcomes, the DA group more frequently considered prophylactic surgery (*P*=0.02) corroborated with higher valuations (*P*=0.04). No differences were found for the other decision related outcomes. Regarding information related outcomes, the DA group felt better informed (*P*=0.00), was more satisfied with the information (*P*=0.00), and showed more accurate risk perceptions. Timing of the DA had no effect on any of the outcomes. No interactions were found between the DA and history of cancer. In conclusion, women being tested for a BRCA1/2 mutation benefit from the DA on information related outcomes. Because timing had no effect, the DA is considered useful either before or after the test result.

The discovery of the BRCA1 and BRCA2 genes has induced widespread interest in genetic testing for inherited susceptibility of breast and ovarian cancer (Miki *et al*, 1994; Wooster *et al*, 1995). Women with a BRCA1/2 mutation have a high lifetime risk for breast cancer (56–85%) and/or ovarian cancer (16–63%) (Easton *et al*, 1995; Struewing *et al*, 1997). They currently face the difficult choice between screening and prophylactic surgery (Burke *et al*, 1997).

An important reason to ask for genetic testing is to obtain certainty about the need for screening and/or prophylactic surgery (Dudok de Wit *et al*, 1997; Lynch *et al*, 1999; Meiser *et al*, 2000). These treatment options have different risk–benefit profiles that women may value differently. The decision about optimal treatment depends on women's personal values for the health states after each of the treatment options (Van Roosmalen *et al*, 2002). In order to choose between screening and prophylactic surgery in a way that reflects their personal values, these women need to be prepared for decision-making by providing information on the treatment options and their risks and benefits.

Decision aids (DAs) are interventions designed to help people make specific and deliberative choices among options by providing information on the options and outcomes relevant to a person's health status (O'Connor *et al*, 2002). Decision aids have been found to be feasible and acceptable to patients and to increase the agreement between patients' values and decisions (Molenaar *et al*, 2000). A recent systematic review found that DAs improve patients' knowledge and realistic expectations of treatment options, reduce decisional conflict, and stimulate patients to play a more active role in decision-making (O'Connor *et al*, 2002). Decision aids appeared to have a variable effect on treatment choice, and little effect on anxiety, satisfaction with the decision-making process and the decision (O'Connor *et al*, 2002). The impact on other outcome measures, such as health outcomes and persistence with treatment choice, remains uncertain (O'Connor *et al*, 2002).

Decision aids in the context of genetic counselling for women already decided to undergo genetic testing for a BRCA1/2 mutation is a new development. There is discussion about the timing of informing women about the treatment options. Some believe that this information should be withheld until after a positive test result in order to prevent unnecessary burden. Others believe that this information should be given earlier to achieve full disclosure of the consequences of a positive test result. Therefore, we investigated the impact of a DA on a broad range of outcomes and also whether the time point of presenting information mattered. The DA consisted of a brochure and video to be viewed at home, providing information on screening and prophylactic surgery, and the physical, emotional, and social consequences. The present study is part of a larger shared decision-making study in which the impact of another DA, including trade-offs and a formal treatment advice derived from a decision model (Van Roosmalen *et al*, 2002), will be evaluated.

## MATERIALS AND METHODS

### Participants

We included women with and without a personal history of breast or ovarian cancer, who provided a blood sample for BRCA1/2 testing at the Family Cancer Clinics of the University Hospitals of Nijmegen (accrual started March 1999), Groningen (accrual started June 1999), and Maastricht (accrual started January 2000). These clinics cover the population of the eastern part of the Netherlands. The closing date for inclusion was November 2001. Women were excluded if they had a cognitive disorder that precluded informed consent, had insufficient knowledge of the Dutch language, were diagnosed with distant metastases, had undergone both bilateral mastectomy and oophorectomy, or had been treated with chemotherapy, radiotherapy, or surgery for breast or ovarian cancer less than 1 month before blood sampling. The study was approved by the local research ethics committees.

### Intervention

The DA consisted of a brochure and video. Unlike usual information material, our information concentrated on contrasting treatment options. The 14-page brochure presented detailed information on treatment options available in 1998 in the Netherlands, and on the physical, emotional, and social consequences in qualitative terms, but whenever possible in quantitative terms (see summary of brochure and references in Appendix A). In the 45 min video, we dealt with the consequences of the treatment options through interviews with eight mutation carriers, with and without a previous history of cancer, who had chosen for either screening or prophylactic surgery. In addition, these women described how they went through the decision-making process. Shots of the results of prophylactic mastectomy with and without a reconstruction were shown. The DA was viewed at home. A short evaluation form was sent with the DA. The video and the evaluation form were to be returned after 1 week. The DA was developed in close collaboration with the specialists involved in the Family Cancer Clinics. It was judged to be balanced in a pretest by the interviewed mutation carriers, the specialists, and the working group on familial cancer of the Dutch Society of Psychosocial Oncology.

### Standard procedure at the family cancer clinics

Genetic testing for a BRCA1/2 gene mutation is offered to women when the family history and the cancer risk estimate suggests a genetic predisposition. Before blood sampling, usually two counselling sessions of 1 h with a geneticist or genetic counsellor take place wherein the family history is discussed, a family pedigree is made, and information is provided on genetic risk, psychosocial consequences of genetic testing, and briefly on the possible treatment options. If the woman decides to undergo genetic testing, a blood sample is obtained. If the mutation is known in the family, an appointment is made for disclosure of the test result after 6–12 weeks. Women without a known mutation in the family receive an invitation for an appointment after extensive molecular analyses, which may take several months.

When a mutation is found, more detailed information is provided on the possible treatment options by a geneticist or genetic counsellor. A social worker or a psychologist is generally present when a positive test result is disclosed to women unaffected with cancer. Mutation carriers are offered additional consultations with a multidisciplinary team involved in the Family Cancer Clinic, generally consisting of a medical oncologist, gynaecologist, and surgeon. These appointments usually take place about 1–2 months after disclosure of a positive test result.

### Randomisation and blinding

Randomisation of the DA took place by family (first-degree up to and including third-degree relatives) to avoid contamination. Randomisation was computer generated in blocks of 10, and stratified by personal medical history of breast/ovarian cancer. Randomisation was performed after obtaining informed consent and the baseline assessment. Neither subjects nor members of the study staff were blinded to intervention assignment.

### Study procedure

**Figure 1:**
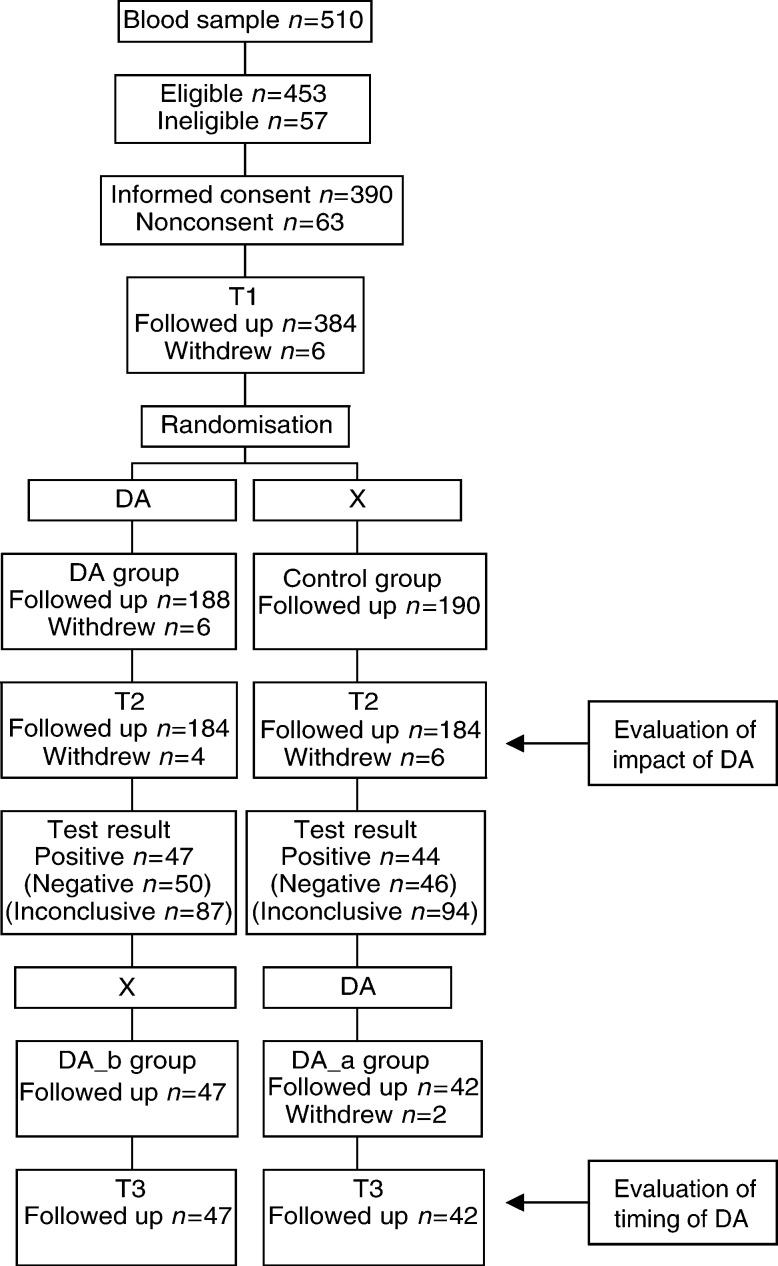
Participant flow.

Eligible women were informed about the present study by the clinical geneticist or genetic counsellor after blood sampling for genetic testing. Women were subsequently contacted by a research assistant to confirm eligibility and to inform them about the study. Women who gave verbal consent were enrolled and received an informative letter describing the study, a consent form, and the baseline questionnaire T1. Women, awaiting their test result, were randomly assigned to the DA group, who received the DA 2 weeks after blood sampling, or to the control group, who received no additional information (see Figure 1

). At 4 weeks after blood sampling (T2), the impact of the DA was evaluated by comparing the DA group with the control group (see Figure 1). Women from the DA group who tested positive (i.e. a BRCA1 or BRCA2 mutation was found) had received the DA *before* the test result (DA_b), and women from the control group subsequently received the DA *after* the positive test result (DA_a). At 2 weeks after a positive test result (T3), the impact of timing of the DA was assessed by comparing the DA_b group with the DA_a group (see Figure 1). Data were collected using questionnaires.

## MEASURES

### Baseline characteristics

Data were obtained on sociodemographic (age, marital status, education level, employment status, presence of children, wanting (more) children, and being religiously affiliated) and medical background (personal and family history of breast/ovarian cancer, time since last cancer diagnosis, whether a mutation was known in the family, whether first-degree relatives had breast or ovarian cancer, and whether they died from breast or ovarian cancer).

### Well-being

We collected data on *anxiety* (state scale of the Spielberger State–Trait Anxiety Inventory) (Spielberger *et al*, 1983), *depression* (Center for Epidemiologic Studies Depression Scale) (Radloff, 1977), and *cancer related distress* (Impact of Event Scale) (Horowitz *et al*, 1979). Furthermore, we asked women to rate their *general health* state during the last week on a 11-point scale ranging from 0 (very bad health state) to 10 (excellent health state).

### Treatment choice

*Treatment choice* was asked for both breasts and ovaries. The treatment choice related to breast cancer risk was between ‘prophylactic mastectomy’, ‘breast cancer screening’, and ‘undecided’. The treatment choice related to ovarian cancer risk was between ‘prophylactic oophorectomy’, ‘ovarian cancer screening’, and ‘undecided’. *Valuations* for the treatment options were asked on a 10-point scale ranging from 1 (very bad) to 10 (excellent).

### Decision related outcomes

The decision related outcomes were asked separately for the breasts and ovaries. An overall score was created by adding the scores for the decision related to breast and ovarian cancer risk and dividing this by the number of items included.

*Strength of treatment preference* was asked on a 4-point Likert scale ranging from 1 (weak preference) to 4 (very strong preference). Those who filled out ‘undecided’ as treatment choice were assigned a value of 0 (no preference).

*Decision uncertainty* was measured with three items related to the uncertainty subscale of the Decisional Conflict Scale by O'Connor (1995). The items were: ‘I doubt what to choose,’ ‘This decision is hard for me to make,’ and ‘I am not sure what to choose,’ measured on a 5-point scale ranging from 1 (very much disagree) to 5 (very much agree).

*Preference for decision-making* was measured with the two decision-making items from the Problem-Solving Decision-Making Scale (PSDM) by Deber *et al* (1996). The items were: ‘Given the risks and benefits of the possible treatment options, who should decide how acceptable those risks and benefits are for you,’ and ‘Given the risks and benefits of the possible treatment options, who should decide which treatment option should be selected,’ measured on a 5-point scale ranging from 1 (doctor alone) to 5 (I alone).

### Information related outcomes

*Subjective knowledge* was measured by asking women to rate their knowledge for prophylactic mastectomy, breast cancer screening, breast self-examination, prophylactic oophorectomy, and ovarian cancer screening, on a 10-point scale ranging from 1 (very bad) to 10 (excellent). An overall score for subjective knowledge was created by adding the scores on the five items and dividing this by five.

*The amount of received information* was measured on a 7-point scale ranging from 1 (I received way too little information), 4 (I received exactly enough information), to 7 (I received way too much information), for the decision related to breast and ovarian cancer risk. An overall score was created by adding the scores and dividing this by two.

*Satisfaction with quality of information* was measured with a 13-item questionnaire on a 6-point scale ranging from 1 (not satisfied), 4 (rather satisfied), to 6 (very much satisfied). Women were offered a series of items regarding cancer risks, efficacy of treatment options, and physical, emotional, and social consequences. An overall score was created by adding the scores on the 13 items and dividing this by 13.

*Risk perception* was asked for the following eight items (the range that we considered accurate for subsequent analyses is given in parentheses): breast (8–14%) and ovarian cancer risk (1–3%) in the general female population, breast (60–85%) and ovarian cancer risk (15–60%) in BRCA1/2 mutation carriers, the possibility of cure when breast (65–80%) and ovarian cancer (35–50%) is detected during screening in BRCA1/2 mutation carriers, and residual risk after prophylactic surgery of breasts (3–12%) and ovaries (3–12%) in mutation carriers. Women were asked to give a risk estimate in a range from 0 to 100%. For each item, a new variable was created classifying the risk estimate as underestimate, accurate, or overestimate. In the analyses, risk accuracy was dichotomised in accurate and inaccurate.

We did not assess the amount of received information, satisfaction with quality of information, and risk perception at baseline to avoid information-seeking behaviour in the control group, thus making the control group less representative.

### Sample size

To detect a difference of at least 10% in the decision uncertainty score between the DA and control groups, with a 5% two-sided significance level and a power of 80%, we needed a sample size of 180 women in each group.

### Statistical analyses

We analysed data from women who completed the questionnaire at baseline and at the time point of interest on an intention to treat basis. In multi-item scales with missing data, scale values were calculated if at least half of the items were filled out by imputing the mean of the remaining items. We compared the intervention groups at baseline using χ^2^ tests for categorical variables, and *t*-tests for continuous variables. To evaluate the impact of the DA, we compared the DA group with the control group at T2 (see Figure 1). To evaluate the impact of timing of the DA, we compared the DA_b group with the DA_a group at T3 (see Figure 1). For continuous variables, comparisons between intervention groups were made using analyses of covariance (ANCOVA) (Maxwell and Delaney, 1990). The baseline assessment of the outcome measure, when present, was included as a covariate. Baseline characteristics tabulated in Table 1

**Table 1:** Baseline characteristics by intervention group

	**DA (*n*=184)**	**Control (*n*=184)**	**DA_b (*n*=47)**	**DA_a (*n*=42)**
*Sociodemographic background*				
Age: mean (s.d.)	43.7 (11.3)	43.5 (10.4)	38.7 (10.0)	40.3 (9.9)
Currently married/partner (%)	84	85	85	86
College or higher (%)	37	32	30	43
Employed (%)	65	65	70	69
Have children (%)	80	83	75	69
Want (more) children (%)	17	15	22	28
Being religiously affiliated (%)	57*	71*	52†	74†
				
*Personal medical history*				
No cancer (%)	46	49	70	76
Breast cancer only (%)	49	46	30	24
Ovarian cancer only (%)	4	4	0	0
Breast and ovarian cancer (%)	1	1	0	0
				
*Family medical history*				
Breast cancer only (%)	47	54	27	20
Ovarian cancer only (%)	3	3	0	3
Breast and ovarian cancer (%)	50	43	73	77
Known familial mutation (%)	47	44	77	76
First-degree relatives with bc or oc (%)	66	69	71	77
First-degree relatives died of bc or oc (%)	28	27	34	31
				
*Well-being*				
Anxiety: mean (s.d.)	40.9 (11.4)*	38.6 (10.7)*	40.1 (10.6)	37.4 (11.5)
Depression: mean (s.d.)	9.3 (8.6)	8.4 (8.1)	7.5 (7.1)	8.6 (8.6)
Cancer related distress: mean (s.d.)	20.1 (14.8)	18.1 (13.5)	18.5 (13.8)	17.1 (12.9)
General health: mean (s.d.)	7.3 (1.7)*	7.6 (1.4)*>	7.9 (1.4)	8.0 (1.5)

bc: breast cancer; oc: ovarian cancer.

* *P*<0.10 comparing the DA and control groups.

† *P*<0.10 comparing the DA_b and DA_a groups.

, which differed between the intervention groups (*P*<0.10), were also included as covariate. Effect sizes (*d*) were calculated as the adjusted mean of the DA group minus the adjusted mean of the control group, and as the adjusted mean of the DA_b minus the adjusted mean of the DA_a group, divided by the standard deviation of the difference score. For two categorical variables, namely ‘treatment choice’ and ‘accuracy of risk perception’, comparisons between groups were made using χ^2^ tests. We used a *P* level of 0.05 to indicate statistical significance. The number of subjects providing data for the various analyses varied due to missing data.

Because randomisation took place by family, and because family members were not independent on the outcome measures, statistical significance will be inflated when all women are treated as independent units. At baseline, we included 33 families with multiple participating members (range 2–5), with a total of 80 women. The subsample of mutation carriers consisted of 10 families with multiple members (range 2–3), with a total of 23 women. To counter inflation, we further examined significant effects by incorporating only the first included family member in the analyses.

In a previous study of ours, we found that after blood sampling for genetic testing, women affected with breast or ovarian cancer reported a worse well-being and a lower preference for participation in decision-making than women without a previous history of cancer (Van Roosmalen *et al*, 2003). To explore a possible differential impact of the DA in women with and without a history of cancer, we examined interactions between the DA (and its timing) and history of cancer using the ANCOVA.

## RESULTS

### Participants

The participant flow is presented in Figure 1. During the study period, 510 women were ascertained of whom 453 (89%) were eligible. Of these 453 women, 390 (86%) gave informed consent. At T1, six (2%) women withdrew. Of the remaining 384 women, 194 were randomised to the DA group and 190 to the control group. Of the 194 women from the DA group, six (3%) withdrew: four indicated an emotional burden related to being informed, one indicated that she did not want to fill out any more questionnaires for emotional reasons, and one indicated that the study was too time consuming. Of the remaining 188 women from the DA group, four (2%) did not view the DA because of an emotional burden. These four women remained in the study and were analysed on an intention to treat basis. At T2, four (2%) women withdrew in the DA group and six (3%) in the control group. In both groups, none of the women declined to receive their test result. In the DA group, 47 (26%) women received a positive test result (38 BRCA1, nine BRCA2). In the control group, 44 (24%) women received a positive test result (28 BRCA1, 14 BRCA2). Mutation carriers from the DA group had received the DA *before* the test result (DA_b). Mutation carriers from the control group subsequently received the DA *after* the test result (DA_a). Of the 44 women from the DA_a group, two (5%) withdrew because of high emotional distress caused by the test result; it is unclear whether their withdrawal was also related to the DA. Of the remaining 42 women from the DA_a group, all viewed the DA. At T3, none of the women withdrew.

### Baseline characteristics

Table 1 presents the sociodemographic and medical background, and the baseline assessment of well-being. Between the DA and control groups, significant differences were found for being religiously affiliated, anxiety, and general health. Between the mutation carriers in the DA_b and DA_a groups, a significant difference was found for being religiously affiliated.

### Evaluation of the decision aid

The DA was viewed once by 49%, and twice or more by 51%. Most respondents (82%) found that it contained exactly enough information, and 13% stated that it contained slightly too little information. In all, 56% reported no negative, 31% a scarcely negative, 12% a rather negative, and 1% a negative emotional reaction towards the information provided. The evaluation forms contained predominantly positive remarks.

### Impact of the decision aid

#### Well-being

No significant differences were found between the DA and control groups for anxiety, depression, cancer related distress, and general health (Table 2

**Table 2:** Impact of DA: unadjusted mean scores (s.d.), results and effect sizes (*d*) from the ANCOVA comparing the DA and C groups

	**Group**	***n***	**T1**	**T2**	**F**	***P***	***d***
*Well-being*							
Anxiety (STAI-state)	DA	176	41.0 (11.4)	40.4 (11.3)			
	C	174	38.3 (10.6)	37.3 (10.6)	1.90	0.17	0.10
Depression (CESD)	DA	176	9.3 (8.6)	8.7 (8.9)			
	C	175	8.1 (7.6)	7.5 (7.3)	0.02	0.89	0.01
Cancer related distress (IES)	DA	169	19.9 (14.7)	18.6 (15.0)			
	C	174	17.6 (13.1)	16.3 (14.2)	0.14	0.71	0.03
General health	DA	173	7.3 (1.7)	7.4 (1.5)			
	C	172	7.7 (1.5)	7.6 (1.6)	0.25	0.62	0.04
							
*Decision related outcomes*							
Valuation of PM	DA	164	5.1 (2.6)	5.6 (2.5)			
	C	160	4.8 (2.5)	5.0 (2.6)	4.41	0.04	0.17
Valuation of BS	DA	165	7.2 (2.5)	6.5 (2.5)			
	C	162	7.5 (2.2)	7.4 (2.1)	14.86	0.00	−0.30
Valuation of PO	DA	161	6.9 (2.3)	7.4 (2.2)			
	C	160	6.7 (2.6)	6.9 (2.5)	4.29	0.04	0.16
Valuation of OS	DA	160	5.9 (2.8)	4.9 (2.5)			
	C	159	6.3 (2.9)	6.0 (2.7)	15.64	0.00	−0.31
Strength of treatment preference	DA	150	2.7 (1.1)	2.6 (1.0)			
	C	150	2.7 (1.1)	2.7 (1.0)	1.38	0.24	−0.10
Decision uncertainty	DA	157	2.7 (1.1)	2.6 (0.9)			
	C	162	2.8 (1.1)	2.7 (1.0)	1.20	0.27	−0.09
Preference for decision-making	DA	155	3.7 (0.6)	3.8 (0.6)			
	C	159	3.5 (0.6)	3.7 (0.6)	1.46	0.23	−0.10
							
*Information related outcomes variables*							
Subjective knowledge	DA	176	6.1 (1.5)	6.8 (1.3)			
	C	174	6.1 (1.5)	6.3 (1.4)	22.31	0.00	0.36
Amount of received information	DA	171	*	3.2 (0.9)			
	C	166	*	2.5 (1.0)	50.37	0.00	0.54
Satisfaction quality information	DA	176	*	3.8 (0.9)			
	C	171	*	3.2 (1.0)	28.48	0.00	0.40

C: control; PM: prophylactic mastectomy; BS: breast cancer screening; PO: prophylactic oophorectomy; OS: ovarian cancer screening;

* not measured at baseline (T1).

).

#### Treatment choice

At baseline (T1), no significant differences were found between the DA and control groups (Table 4). At T2, significant differences were found; women in the DA group more often chose for prophylactic surgery. For the valuation of the treatment options (Table 2), significant differences were found between the DA and control groups; women in the DA group gave a higher value for prophylactic surgery and a lower value for screening, corroborating our finding of more choices for prophylactic surgery.

#### Decision related outcomes

For strength of treatment preference, decision uncertainty, and preference for decision-making (Table 2), no significant differences were found.

#### Information related outcomes

For subjective knowledge, amount of received information, and satisfaction with quality of information (Table 2), significant differences were found between the DA and control groups; women in the DA group felt better informed and were more satisfied with the amount and quality of the information.

The mean risk estimates, the percentage under-, accurate, and overestimates, are presented in Table 5. Significant differences were found in risk accuracy for three of the eight items; the DA group made significantly more accurate risk estimates for BRCA1/2 related ovarian cancer risk, and cure of BRCA1/2 related breast cancer diagnosed during screening and cure of BRCA1/2 related ovarian cancer diagnosed during screening.

### Impact of timing of the decision aid

For well-being, decision and information related outcomes (Table 3

**Table 3:** Impact of timing of DA: unadjusted mean scores (s.d.), results and effect sizes (*d*) from the ANCOVA comparing the DA_b and the DA_a groups

	**Group**	***n***	**T1**	**T3**	**F**	***P***	***d***
*Well-being*							
Anxiety (STAI-state)	DA_b	45	40.3 (10.8)	40.9 (12.9)			
	DA_a	40	37.8 (11.5)	40.2 (12.4)	0.25	0.62	−0.08
Depression (CESD)	DA_b	45	7.7 (7.2)	11.2 (11.0)			
	DA_a	39	8.6 (8.8)	9.4 (9.4)	2.42	0.12	0.24
Cancer related distress (IES)	DA_b	44	18.4 (13.5)	22.6 (15.7)			
	DA_a	40	17.3 (13.1)	20.3 (13.9)	0.36	0.55	0.09
General health	DA_b	43	7.8 (1.5)	7.4 (1.9)			
	DA_a	37	8.0 (1.5)	7.7 (1.7)	0.23	0.63	−0.08
							
*Decision related outcomes*							
Valuation of PM	DA_b	44	5.0 (2.4)	5.6 (2.5)			
	DA_a	39	4.0 (2.4)	4.7 (2.7)	0.02	0.89	0.02
Valuation of BS	DA_b	44	7.3 (2.4)	6.3 (2.3)			
	DA_a	39	8.2 (1.6)	7.4 (2.1)	1.16	0.29	−0.17
Valuation of PO	DA_b	41	6.7 (2.7)	7.3 (2.6)			
	DA_a	38	5.8 (2.8)	6.5 (2.5)	0.09	0.77	0.05
Valuation of OS	DA_b	41	5.6 (2.9)	4.7 (2.8)			
	DA_a	38	7.2 (2.5)	6.2 (2.8)	0.80	0.38	−0.14
Strength of treatment preference	DA_b	40	2.8 (1.2)	3.1 (0.8)			
	DA_a	36	2.6 (1.1)	2.8 (1.0)	0.64	0.43	0.13
Decision uncertainty	DA_b	43	2.5 (1.1)	2.1 (0.9)			
	DA_a	38	2.7 (1.1)	2.4 (1.0)	0.29	0.59	−0.08
Preference for decision-making	DA_b	42	3.8 (0.6)	3.8 (0.7)			
	DA_a	37	3.5 (0.6)	3.6 (0.7)	0.17	0.69	−0.06
							
*Information related outcomes variables*							
Subjective knowledge	DA_b	45	6.2 (1.8)	7.1 (1.3)			
	DA_a	40	6.1 (1.5)	6.8 (1.4)	0.83	0.37	0.14
Amount of received information	DA_b	43	*	3.3 (0.9)			
	DA_a	40	*	3.3 (0.9)	0.25	0.62	0.08
Satisfaction quality information	DA_b	45	*	3.9 (1.0)			
	DA_a	38	*	3.7 (0.9)	1.01	0.32	0.15

PM: prophylactic mastectomy; BS: breast cancer screening; PO: prophylactic oophorectomy; OS: ovarian cancer screening;

* not measured at baseline (T1).

), and treatment choice (Table 4

**Table 4:** Treatment choice related to breast and ovarian cancer risk at T1 and T2 (for the DA and control groups) or T3 (for the DA_b and DA_a groups)

	**DA**	**Control**	**DA_b**	**DA_a**
*Treatment choice related to bc risk at*	*T1*		*T1*	
Prophylactic mastectomy	61 (35)	53 (31)	17 (37)	8 (20)
Breast cancer screening	92 (53)	103 (60)	24 (52)	30 (73)
Undecided	20 (12)	15 (9)	5 (11)	3 (7)
	*χ*^2^=1.89; *P*=0.39		*χ*^2^=4.13; *P*=0.13	
				
*Treatment choice related to bc risk at*	*T2*		*T3*	
Prophylactic mastectomy	69 (41)	55 (32)	18 (40)	8 (21)
Breast cancer screening	80 (47)	104 (61)	26 (58)	29 (74)
Undecided	21 (12)	11 (7)	1 (2)	2 (5)
	*χ*^2^=7.84; *P*=0.02		*χ*^2^=3.94; *P*=0.14	
				
*Treatment choice related to oc risk at*	*T1*		*T1*	
Prophylactic oophorectomy	112 (66)	105 (62)	27 (61)	21 (51)
Ovarian cancer screening	36 (21)	44 (26)	10 (23)	15 (37)
Undecided	21 (13)	20 (12)	7 (16)	5 (12)
	*χ*^2^=1.05; *P*=0.59		*χ*^2^=1.98; *P*=0.37	
				
*Treatment choice related to oc risk at*	*T2*		*T3*	
Prophylactic oophorectomy	122 (73)	107 (63)	34 (81)	22 (56)
Ovarian cancer screening	24 (14)	48 (28)	6 (14)	14 (36)
Undecided	21 (13)	14 (8)	2 (5)	3 (8)
	*χ*^2^=10.37; *P*=0.01		*χ*^2^=5.87; *P*=0.05	

bc: breast cancer; oc: ovarian cancer. Values are numbers (percentage) of women.

), no significant differences were found between the mutation carriers in the DA_b and DA_a groups, with the exception that the DA_a group made significant more accurate risk estimates for cure of BRCA1/2 related ovarian cancer diagnosed during screening (Table 5

**Table 5:** Mean risk perception (s.d.), percentage under- (−), accurate (=), and overestimates (+) by intervention group

		**T2**					**T3**			
	**Group**	**Mean (s.d.)**	**−(%)**	**=(%)**	**+(%)**	**Group**	**Mean (s.d.)**	**−(%)**	**=(%)**	**+(%)**
*General*										
bc risk	DA	25.3 (17.4)	2	41	57	DA_b	20.4 (18.6)	9	56	35
	C	27.0 (19.8)	3	38	59	DA_a	21.0 (18.8)	5	59	36
oc risk	DA	20.3 (17.9)	1	11	88	DA_b	16.4 (21.0)	2	24	74
	C	21.1 (18.4)	2	7	91	DA_a	11.9 (12.8)	0	21	79
										
*BRCA1/2*										
bc risk	DA	65.2 (17.4)	40	57	3	DA_b	70.6 (14.7)	28	68	4
	C	62.8 (19.2)	43	53	4	DA_a	69.5 (15.3)	27	70	3
oc risk*	DA	55.6 (18.9)	3	65	32	DA_b	52.6 (17.7)	2	73	24
	C	55.5 (21.7)	7	54	39	DA_a	48.7 (20.5)	5	72	23
Cure bc*	DA	60.4 (21.3)	49	43	8	DA_b	61.9 (24.2)	39	50	11
	C	56.2 (12.4)	59	32	9	DA_a	68.0 (17.1)	34	58	8
Cure oc*†	DA	44.7 (22.0)	33	38	37	DA_b	37.9 (26.7)	48	24	28
	C	45.3 (26.9)	39	24	29	DA_a	40.4 (22.4)	32	47	21
bc risk after PM	DA	10.3 (15.0)	37	43	20	DA_b	8.1 (9.3)	41	41	18
	C	15.8 (20.8)	30	38	32	DA_a	8.7 (9.4)	38	42	20
oc risk after PO	DA	8.7 (14.4)	50	32	18	DA_b	6.3 (9.3)	48	39	13
	C	13.0 (20.5)	41	32	27	DA_a	6.7 (6.8)	45	38	17

C: control; bc: breast cancer; oc: ovarian cancer; PM: prophylactic mastectomy; PO: prophylactic oophorectomy.

* *P*<0.05 for risk accuracy comparing the DA and control groups.

† *P*<0.05 for risk accuracy comparing the DA_b and DA_a groups.

).

### Additional analyses incorporating only the first included family member

Significant differences found above were further tested by including only the first family member. Differences between the DA and control groups were no longer significant for the valuation of prophylactic mastectomy (*P*=0.13), the valuation of prophylactic oophorectomy (*P*=0.15), and risk accuracy of BRCA1/2 related ovarian cancer risk (*P*=0.18).

### Interaction between the decision aid (and its timing) and history of cancer

No interactions were found between the DA and history of cancer. No interactions were found between timing of the DA and history of cancer, except for valuation of prophylactic mastectomy (*P*<0.01) and breast cancer screening (*P*<0.01). Women affected with cancer valued prophylactic mastectomy higher and breast cancer screening lower in the DA_a group compared to the DA_b group, whereas for women without a previous history this was in the opposite direction.

## DISCUSSION

The present study is the first to evaluate the impact of a DA and its timing in women being tested for a BRCA1/2 mutation. Both women with and without a previous history of cancer were included. The brochure and video were designed to help women make informed decisions, and concentrated on contrasting the various risks and benefits of screening and prophylactic surgery and the physical, emotional, and social consequences. The DA had no impact on well-being. The DA led to more considerations towards prophylactic surgery and corroborating higher valuations for prophylactic surgery. The DA had no impact on strength of treatment preference, decision uncertainty, and preference for decision-making. The DA improved information related outcomes. In general, timing of the DA had no impact on any of the outcome measures. No interactions were found between the DA and history of cancer.

Previous studies on genetic testing for a BRCA1/2 mutation did not find substantial psychological morbidity among women initiating genetic testing or receiving a positive test result (Lerman *et al*, 1996, 1998; Croyle *et al*, 1997; Lodder *et al*, 1999, 2001; Coyne *et al*, 2000; Schwartz *et al*, 2002), which is in agreement with the well-being levels in our study. The DA had no negative impact on a broad range of well-being outcomes, and did not deter women from receiving their test result, while it improved understanding of the treatment options and consequences. Similar results on the use of a video in BRCA1/2 counselling have been reported (Cull *et al*, 1998). Their video, however, was more general and introductory and less focused on the treatment decision. Another difference is that it was provided either before or after the first genetic counselling session, whereas our information was provided after a blood sample was taken or after a positive test result.

Women in the DA group were more inclined towards prophylactic surgery. This was surprising as the DA was judged to be balanced in a pretest. It also described the negative consequences of prophylactic surgery in words and pictures. The trend of the decision towards prophylactic surgery, while interesting, is not a valid criteria for judging the DA. Therefore, a more relevant question is whether the DA led to a reduction of decision uncertainty, and whether it stimulated a preference for a more active role in decision-making; our results did not show such benefits despite the fact that women felt better informed about the treatment options. As our DA was simple and was to be viewed at home, that is without face-to face support, a more intensive DA might prove more effective (O'Connor *et al*, 2002).

The largest and most consistent benefits of DAs are better knowledge and more realistic expectations of treatment options (O'Connor *et al*, 2002). We also found that women felt better informed, were more satisfied with the amount and quality of the information, and had more accurate risk perceptions, after viewing the DA. A clear marker of the information need is that most women viewed the DA, and about half of the women viewed the DA twice or more. Only a few women did not want to see the DA while awaiting their test result. Despite improvements in risk perception, on average 60% of women in the DA group still had risk perceptions that were inconsistent with a broadly defined range of accuracy. Further research is needed to enhance risk perception. The positive effects of our DA occurred irrespective of whether it was presented before or after the test result.

The strength of our study is that we included a consecutive sample of women covering the eastern part of the Netherlands. Some limitations should be considered. First, we did not evaluate the impact of timing of the DA in women receiving an inconclusive test result (*n*=181). This was beyond the scope of our study, which focused on mutation carriers subsequently. Second, the subsample of mutation carriers is relatively small reducing the power to detect meaningful differences between the women who received the DA either before or after the test result. Third, all treatment choices, even those obtained 2 weeks after a positive test result, are merely intentional as prophylactic surgery is usually postponed for several months until all specialists have been consulted or even longer in young women. However, additional clinical follow-up showed that intended treatment choices, obtained shortly after a positive test result (T3), are strongly predictive of the executed treatment at 9 months after a positive test result: for example, 53% of the women opting for prophylactic mastectomy had undergone this treatment, compared to none of the women opting for breast cancer screening. Fourth, long-term follow-up on the effects of the DA was not obtained because all mutation carriers eventually had received the DA. In the second part of our study, evaluating another DA, including trade-offs and a formal treatment advice derived from a decision model (Van Roosmalen *et al*, 2002), the follow-up of these mutation carriers will be continued. The brochure was based on the best knowledge available at that time and needs regular updates. Our results showed that it does not matter whether the DA is given to women before or after disclosure of the BRCA1/2 test result. However, in the waiting period before the test result, women do benefit from the DA on information related outcomes such as subjective knowledge, satisfaction with the amount and quality of information, and risk perception. Only few women may prefer to postpone the DA until being tested positive. Positive effects occurred irrespective of history of cancer; thus the DA is considered useful both for women with and without a previous history of cancer. Therefore, our advice is to offer the DA, in addition to genetic counselling, to all women on a voluntary basis after taking the blood sample, while making clear that the information is also available after the test result.

## Appendix A: Summary of information presented in the brochure

**Introduction**
*Aim of information material*: supplementary information to genetic counselling.
*General information:*: procedures at the Family Cancer Clinic, patterns of inheritance, outline of choices.
**Breast cancer**
*Sporadic breast cancer*: 11% lifetime risk (Netherlands Cancer Registry, 1995), mostly occurs after the age of 50, general risk factors.
*Hereditary breast cancer*: 35–50% risk before age 50, 60–85% lifetime risk (Easton *et al*, 1995; Struewing *et al*, 1997), characteristics of hereditary breast cancer.
**Breast cancer screening**
*Efficacy*: 80% of breast cancers detected in early stage during screening, 70–75% of these breast cancers are curable (Smart *et al*, 1997). Prognosis about the same as sporadic breast cancer (Verhoog *et al*, 1998), not everybody will develop breast cancer during screening.
*Procedure*: generally from age 25, monthly breast self-examination, 6-monthly physical examination by a physician, yearly mammography.
*Additional information*: surgery for breast cancer, future developments, MRI, biopsies.
*Psychosocial consequences*: more awareness of physical symptoms of the body sometimes may lead to more physical complaints, worse psychological well-being in some women due to fear of cancer, which may affect marital relationships. Better coping with the situation over time.
**Prophylactic mastectomy**
*Efficacy*: small remaining risk of breast cancer (Hartmann *et al*, 1999).
*Procedure*: about 1 week admission in hospital, healing of wound takes some time, mostly without complications, no lymphoedema.
*Additional information*: breast reconstruction and alternatives, consequences and complications.
*Psychosocial information*: mostly a deliberated choice and therefore better accepted, feelings of relieve, negative impact on body image and sexuality in some women. Better cooping with situation over time.
**Ovarian cancer**
*Sporadic ovarian cancer*: 1.8% lifetime risk (Netherlands Cancer Registry, 1995), mostly occurring at age 45–60, general risk factors.
*Hereditary ovarian cancer*: BRCA1 40–60% lifetime risk, BRCA2 15–20% lifetime risk, low risk before age 40, sharp increase from age 40 (Easton *et al*, 1995; Struewing *et al*, 1997).
**Ovarian cancer screening**
*Efficacy*: efficacy unproven, 25% of ovarian cancer detected in early stage in unscreened population (Ries *et al*, 1973–1998), 40–45% of hereditary ovarian cancer is curable, indication of better prognosis for hereditary as compared to sporadic ovarian cancer (Rubin *et al*, 1996), not everybody will develop ovarian cancer during screening.
*Procedure*: generally from age 35, yearly transvaginal ultrasound, gynaecologic examination, CA125 testing.
*Additional information*: future developments, biopsies, surgery for ovarian cancer.
*Psychosocial consequences*: more awareness of physical symptoms of the body sometimes may lead to more physical complaints, worse psychological well-being in some women due to fear of cancer, which may affect marital relationships. Better cooping with the situation over time.
**Prophylactic oophorectomy**
*Efficacy*: small remaining risk of ovarian cancer (Piver *et al*, 1993).
*Procedure*: mostly around age 40, about 2 days admission in hospital, usually laparoscopic surgery with minimal morbidity, after surgery ongoing CA125 control.
*Additional information*: menopause and consequences (increased risk for cardiovascular diseases and osteoporosis), hormone replacement therapy and consequences.
*Psychosocial information*: most complaints due to effect of menopause.

### APPENDIX REFERENCES

Easton DF, Ford D, Bishop DT, the Breast Cancer Linkage Consortium (1995) Breast and ovarian cancer incidence in BRCA1-mutation carriers. *Am J Hum Genet* **56**: 265–271

Hartmann LC, Schaid DJ, Woods JE, Crotty TP, Myers JL, Arnold PG *et al* (1999) Efficacy of bilateral prophylactic mastectomy in women with a family history of breast cancer. *N Engl J Med* **340**: 77–84

Netherlands Cancer Registry (1995) *Incidence of cancer in the Netherlands*. Fourth report of the Netherlands Cancer Registry. Utrecht, The Netherlands

Piver MS, Jishi MF, Tsukada Y, Nava G (1993) Primary peritoneal carcinoma after prophylactic oophorectomy in women with a family history of ovarian cancer. A report of the Gilda Radner Familial Ovarian Cancer Registry. *Cancer* **71**: 2751–2755

Ries LAG, Eisner MP, Kosary CL *et al* (eds) *SEER cancer statistics review, 1973–1998*. Bethesda, MD: National Cancer Institute

Rubin SC, Benjamin I, Behbakht K, Takahashi H, Morgan MA, LiVolsi VA, Berchuck A, Muto MG, Garber JE, Weber BL, Lynch HT, Boyd J (1996) Clinical and pathological features of ovarian cancer in women with germ-line mutations in BRCA-1. *N Engl J Med* **335**: 1413–1416

Smart CR, Byrne C, Smith RA, Garfinkel L, Letton AH, Dodd GD, Beahrs OH (1997) Twenty years of follow-up of the breast cancers diagnosed during the breast cancer detection demonstration project. *Ca Cancer J Clin* **47**: 134–149

Struewing JP, Hartge P, Wacholder S, Baker SM, Berlin M, McAdams M, Timmerman MM, Brody LC, Tucker MA (1997) The risk of cancer associated with specific mutations of BRCA1 and BRCA2 among Ashkenazi Jews. *N Engl J Med* **336**: 1401–1408

Verhoog LC, Brekelemans CTM, Seynaeve C (1998) Survival and tumour characteristics of breast-cancer patients with germline mutations of BRCA1. *Lancet* **351**: 316–321
